# Importance of Physical Signs

**Published:** 1836

**Authors:** 


### VII.—Importance of Physical Signs.

*A Dissertation on the Importance of Physical Signs in the various Diseases of the Abdomen and Thorax.* By Robert W. Haxall, M. D., of Richmond, Va. Boston: Perkins & Marion, 1836.

The above is the title page of an essay of 108 pages which was placed in the hands of the editors of the Journal a few weeks since. The writer it seems was the successful competitor for the premium offered by the Boylston Medical committee, a year or more since, for the best essay "on the importance of physical signs in the various diseases of the abdomen and thorax." Although it contains but little that may not be found in the books already published, it places the author before the public as a man of some learning and research.

Much of the material of which the dissertation is composed is drawn from European writers, especially the French, whose zeal and industry in the prosecution of pathological anatomy has given them the advantage of others in the knowledge of this important department. Although the writer informs us that he has, with few exceptions, commented on no condition of disease which he has not witnessed in life or in death, still one of the greatest faults of modern writers, and especially American Essayists, is the inclination to follow in the leading strings of great names without investigating for themselves. If every physician entered, like Hunter, upon the examination of the principles of his profession with a determination to take nothing upon trust within the reach of positive proof, our knowledge would be much more extensive than it is at present. The writer of the dissertation before me may be of this class, but still I must confess that I felt disappointed upon a perusal of the work. Little or nothing is said of the skin, the appearance of the surface of the body, the expression of countenance, the state of secretions and excretions, in the course of the essay, notwithstanding all these afford much that is valuable in the physical symptoms of disease in the great abdominal and thoracic cavities. It is also evident that a strict observation of the diseases in different climates, would reveal much to us that is peculiar of each. In hepatic derangements, for instance, the physical signs would vary according to the location of the patient in the northern or southern portions of our country, and so of many other visceral affections. I may be told, however, that the terms of the premium did not admit of a thorough investigation of the various physical signs of disease in the abdomen and thorax, but only of their importance, to a correct diagnosis, admitting that they are already known: but little is said in the essay to impress this upon the profession. No cases are given to illustrate the danger of neglecting such symptoms, or the great importance of attending to them in our clinical researches. The writer also seems anxious to swell many of his physical signs into importance by observations and details which cannot be relied upon as affording correct evidence, in many cases, at least, which have fallen under my notice. The tumefaction of the liver, the inequalities of its surface, the evidence of percussion, the comparative density of a variety of internal organs, etc., are of this class. It would certainly be extremely difficult to determine the inequalities of the surface of the liver, or even the softening of portions of it through the parieties of the abdomen, especially when we consider that it occupies to a great extent, the concavity of the diaphragm and is, of course, surrounded in part by a bony covering. It would also require an experienced ear to determine the difference of resonance in percussion over an abscess, or general softening, or even an enlargement of this organ from the simple congestion mentioned by the writer. But as his entire remarks on the liver may be interesting to the readers of the Journal, they will be found in the following extract. Notwithstanding the diseases of our valley are said to be principally billious I have rarely seen an unhealthy liver upon post mortem examination, even when that organ was supposed to be affected, previous to the death of the patient. Such derangements must therefore consist in lesions of function rather diseases of structure.

" *The Liver.*—We should not probably deviate far from the truth in making the assertion, that a greater obscurity attends the various diseases of this viscus, than of any other abdominal organ, except perhaps the spleen. That a knowledge however, of its functional symptoms can avail us nothing, we not only do not say, but admit that it is frequently necessary in conjunction with the physical signs to the formation of a clear diagnosis. Nay, we will even go further, and say that the union of the two cannot always enlighten us as to the exact affection which may exist.

For several years we have entertained the opinion founded upon the observation of many cases, that there was in the whole range of functional symptoms but one which had an invariable and permanent existence;—we mean the appearance of jaundice, which is only *constant* where there exists some obstruction in the ducts. In all the other presumed affections we have noticed, it has by no means been a diagnostic or even a permanent symptom, for we have seen active and chronic congestion with it and without it; and the same remark may be made in relation to hypertrophy and atrophy, hydatids, cancerous and fatty degenerations, induration and abscess. It is certainly impossible to offer a positive explanation of the existence or non-existence of jaundice in these different affections, in the present state of the science; and a similar position may be advanced in reference to the discoloration of the feces and the yellow appearance of the urine. Much yet remains to be accomplished by patient and repeated observation both during life and after death.

Nor can we always speaks positively as to the location of *pain* which sometimes exist and at others does not; and when it does, we cannot be entirely certain that some partial peritonitis in the immediate neighborhood of the liver, or inflammation of the diaphragmatic pleura, or rheumatic affection of the abdominal muscles, may not exist as the true lesion. In inflammation of the pleura lining the diaphragm we are in truth subjected to a still farther deception, for cases have occurred where jaundice has been added to the train of symptoms, arising beyond doubt from sympathetic irritation. Misapprehension may also arise by referring the seat of pain to the liver, when in reality it has its existence in an inflamed pylorus or duodenum.

Having thus stated the ambiguity which attends the two most prominent functional symptoms in the diseases of the liver, we shall proceed to designate a few, and endeavor to point out that combination of general and physical signs which may lead to a clearer diagnosis than could be derived from a separate consideration of the one or the other.

In simple inflammatory engorgement whether active or passive (chronic,) we may or we may not have all the functional symptoms, as pain, jaundice, discoloration of the feces, and coloration of the urine; (I make no mention of a coated tongue and fever, inasmuch as they are common to nearly every disease to which the human frame is liable.) And should every one of these symptoms supervene, a combination which does not always happen, how do we certainly know that this particular disease exists, when each one has been observed in some different affection? We must resort then to some other sign, and we find it in the abnormal peculiarity which both percussion and palpation presents. This peculiarity takes place in consequence of the distended viscus passing beyond its accustomed limit into portions of the abdomen where it does not naturally belong, and by the sensation which we are able to recognize by the touch. It is astonishing to notice the extent to which this enlargement may occasionally proceed. Andral relates a case where the liver was found occupying not only the epigastric, but a portion of the left hypochondriac region and extending to within a short distance of the crest of the right iliac bone.

The tumor formed in simple active or passive engorgement is found to be smooth, without prominence or depression, and in consequence of its affording a dull or flat sound on percussion, we may thus measure the extent to which it has attained. We know the regions occupied by the stomach and the ascending colon; and we know too that as they are hollow organs, percussion will elicit a certain resonance peculiar to each one; now, if this resonance is replaced by a dull and flat sound, and we are enabled by the touch to trace a continuous enlargement beneath the cartilages of the ribs, we have every reason to ascribe it to an altered condition of the liver. Whenever too the tumor exists to any considerable degree, there is an evident alteration in the *form* of the abdomen; the ribs are rendered more prominent, as well as that portion of the abdominal surface situated to the left and below them.

A general enlargement of this organ is not uniformly the result of those causes which produce hepatitis; for numerous autopsies have demonstrated the fact that one or the other lobe may be separately engorged, and so far as the left lobe is implicated, the local signs which have been enumerated will suffice. The right lobe may be alone engorged and distended to such a degree as considerably to impinge against the diaphragm, and thereby cause an interference with the free action of the lung; and from this source we derive one of our diagnostic signs, for the application of the stethoscope will reveal to us an absence of respiration in that portion of the chest, where it ought to exist; and percussion will furnish another proof in the pressence of a dull and flat sound where it ought not to be. It may perhaps be objected to each of these positions, that a similar result might be produced by a different complication, as for example by a pleuritic effusion; but a little attention it seems to us will cause the difficulty to vanish. When effusion has taken place, there is, it is true, a dull sound upon percussion and an absence of respiration; but we have other symptoms, as will be shown when we come to speak of diseases of the chest; and we ought not to lose sight also of the fact, that the primary symptoms of pleurisy could hardly have been mistaken by the physician or forgotten by the patient. Besides, the dullness of sound produced by effusion will be found by no means so intense, as that occasioned by the presence of an engorged liver.

There is another condition where the liver forms a considerable prominence, not only in the epigastrium, but even in the left hypochondrium, and yet it may not be in the least diseased. This deceptive form of enlargement results either from a very copious effusion into the cavity of the right pleura, or from the growth of some abdominal tumor pushing it beyond its proper limits. In addition to the physical signs developed upon percussion and auscultation, of a pleuritic effusion, there is an absence of any functional symptom which might mark a disease belonging properly to the liver itself; and we are thus enabled, from a union of these two considerations, to arrive at a conclusion approaching to certainty in our diagnosis. Should the hepatic prominence be the effect of the second cause we have just mentioned, much more difficulty we admit may arise, and thus render the case a doubtful one; but yet not invariably, for there are instances where the tumor may be distinctly traced beneath the protruded liver, while the epigastric border of this organ may be felt reposing upon it, and not reaching to the entire extent of the tumor itself.

Another variety of hepatic engorgement is occasionally met with, which upon its first occurrence at least, or even when often repeated, is the result of a purely mechanical cause; it arises from an obstruction to the equable flow of the blood through the hepatic veins, in consequence of disease of the right side of the heart. In its inception, as we have said, this engorgement acknowledges only a mechanical cause, and so long as the vital actions remain undisturbed, we notice no sympathetic febrile affection; and an additional peculiarity may be remarked, which if it be not pathognomonic, will at least lead us to examine into the heart's functions. We allude to the *inter-*

*mission* so frequently noticed in this form of congestion. For a day or two, or longer, the liver remains more or less enlarged, exhibiting none, or a scarcely appreciable deviation in the healthy play of its functions, and then unexpectedly returns to its nomal dimensions. From the frequency however with which this occurs, its vital actions may become implicated, irritation supervenes, is prolonged by the engorgement continuing longer than usual, and finally inflammation with all its characteristics forms the last link in the chain. Patients have died from the complication just described, and without a knowledge of the heart's disease, the wonder has been what *miasmatic* influence, or what sudden impression of cold when every care had been so assiduously taken to guard against it, could have produced a hepatitis. Although somewhat foreign to our subject, we may here be allowed to remark, that hepatic congestion in one or the other of its forms, is very often consecutive to an acute or chronic duodenitis. Many autopsies have demonstrated this fact, and unless we very carefully analyze and understand the symptoms peculiar to each, we may be led into a double error; the one an error of diagnosis, and the other of treatment. The uncertainty as to the proper location of pain even when augmented upon pressure, and the early appearance of icterus and whitish evacuations lead us to locate the lesion solely in the hepatic organ; and dose after dose of some drastic cathartic, while it fails to cure the consequent, cannot do otherwise than augment the antecedent disease. Ere long the nervous centres become involved, the pulse is hurried and compressible, the teeth are fuliginous; the tongue is hard and blackened or shining and furrowed; stimulant after stimulant is poured down upon viscera which *cannot* react in consequence of the overwhelming inflammation which these very remedies have produced, and death finally comes to the relief of the unfortuate patient. Is this an overwrought picture? By no means. How many have been taught to see in this catenation of symptoms the true evidences of *debility*, and how many will bear witness to the mournful truth that they have seen their patients sink time after time in despite of bark, and wine, and brandy. That they do not always sink, is owing to the gratifying circumstance that nature will sometimes conquer both the disease and the physician.

If we expect to draw an indication as to the positive nature of many other affections of the liver from its functional aberrations, we shall find ourselves exposed to uncertainties, similar to those already cited. We will therefore endeavor to point out a few local signs which may assist us to form a proper opinion.

The liver consists in its general structure of two distinct formations; the one whitish substance of considerable density, inclosing and supporting blood-vessels of various diameters, but without permitting their indefinite and diversified ramifications; the other substance is a red and highly vascular parenchyma, situated within the interstices of the first, and eminently capable of undergoing a change either in the diminution or augmentation of its volume. When we make a section of the organ in a state of health the peculiar structure we have indicated may be recognized, but much more clearly however in a diseased condition. Either one of these formations may be separately affected; in the one we may discover hyperthrophy of its tissue, while that of the other remains undisturbed; and vice versa; or the one may be subjected to an increase in its nutrition, while the other suffers a diminution, or both may be simultaneously diseased in whole or in part.

We have recurred to this feature in the structure of this viscus, for the purpose of explaining more satisfactorily, the peculiarity which its hypertrophy gives to palpation, when carried to an extent sufficient to produce a tumor. The touch then recognizes a tumor of greater or less size, presenting manifest *inequalities*, in consequence of every portion of the organ not being similarly hypertrophied; the white or red substance being alone altered, or both in unequal degrees. Hypertrophy is rarely or never met with as an idiopathic affection but may be considered the result of an inflammation primitively chronic. From this circumstance we can scarcely confound it with active engorgement, leaving out of the question the assistance we are enabled to derive from local signs; for there is an absence of those sympathetic disturbances which almost invariably characterize that affection.

The morbid increase of the nutritive function expressed by the term hypertrophy, being the result of primitive chronic engorgements, may exist as such for a longer or shorter period; but it is frequently noticed as the second link in that chain which ultimately terminates in disorganization, and autopsy reveals the existence of fatty substances, tubercular excavations, and cancerous degenerations, depending doubtless upon constitutional idiosyncracy. If we have had an opportunity of following the case from its commencement, and if we have noticed the inequalities upon the surface of the tumor, we shall by and by find that they have grdually disappeared, wholly or partially, and been substituted by well-marked depression; and from this local sign we draw the conclusion that softening or ulceration has supervened, the final step in the process of disorganization. That a cancerous affection of the liver may be primitive, there is abundant evidence to believe; and when it forms a perceptible enlargement, its surface will present to the touch numerous corrugations or small prominences which finally terminate in ramollissment. Here then the physical signs present a similarity in this disease and tuberculous degenerescence, and tend very much to obscure the diagnosis; and although it may often be impossible to speak assuredly, yet may we derive some assistance from the character of the *pain* which accompanies the two affections; in the former it is acute and lancinating, while in the latter it is rather a sense of indescribable uneasiness, amounting but seldom to positive pain.

Abscess of the liver may be the result of external violence, of injuries inflicted upon the head, or of active inflammation produced by some other cause. We recognize this condition by the various signs heretofore mentioned; and should shivering sensations be experienced towards the close of the acute stage of hepatitis, observation authorizes the conclusion that an abscess has formed. If it be deeply seated, there may be no perceptibly circumscribed tumor, and the general enlargement may present to the touch all the characters of simple engorgement. When, however, it becomes more superficial, the tact of the skilful experimenter will, if he cannot detect a fluctuation, very clearly discover a *yielding* surface beneath his fingers, surrounding which he will mark the hard, and dense, and unaltered substance of the liver. Besides this, percussion will here come in to our aid, and the difference in the resonance will denote the extent of the purulent collection; the sound being duller over the healthy portions of the viscus, in consequence of its greater density.

From numerous autopsies made at La Pitie under the direction of M. Andral, in hepatic diseases, where dropsy has been the result, the fact has been ascertained, that effusion much more frequently succeeds to a diminution or atrophy of the organ than to any other affection. The diagnosis is rendered much more obscure in consequence of the very frequent absence of functional symptoms. A local examination, when the abdomen is not excessively distended, may lead us to suspect the true lesions by observing that the liver does not reach its normal dimensions; and if the patient be placed in an erect position, we can discover an unnatural increase of resonance upon percussion. We may also be assisted in our efforts at a correct opinion, by questioning the functions and physical condition of other organs, the lesions of which may result in effusion. Has the ascites been produced by disease of the heart? We recognize by means of the stethoscope its healthy condition; and we know too that when effusion has followed as a sequence to its disease, its very first appearance is discoverable around the malleoli; and it is not until some time has elapsed, that upper portions of the inferior members and the abdomen become implicated; or, has it been the effect of peritonitis in either of its forms? Neither the patient or his attendant could have mistaken the earlier symptoms which so well characterize the inflammation of this serous membrane. By thus excluding all other organs which may be found in a state of health, or upon the morbid condition of which, such and such symptoms could not depend, we may be enabled to locate the lesion by inference at least, and address our remedies to the proper point. It may not be unimportant to state, that a similar method of investigation will frequently avail much in other affections.

The various tunics which enter into the composition of the gall-bladder, the cystic and hepatic ducts, and the choledochus common to the two, are liable to the same morbid alterations that are discoverable in other organs; and these tissues may be separately or simultaneously diseased. The functional symptoms of icterus and discoloration of the feces, are constant in some of these lesions, but yet are incapable in themselves alone, of indicating the exact affection; some of their diseases too, with the aid both of general and local symptoms are recognizable only after death. Whatever cause, operating mechanically, produces an obliteration or offers an obstruction to the free course of the ductus choledochus, will give rise to the two symptoms. With respect to those causes which are *purely* mechanical, and which act in no other way than by obstructing the canal, may be classed the different varieties of calculus, whether primitive in their formation, or the result of the inspissation of the ingredients which enter into the composition of the bile. The general and functional symptoms dependent upon this condition are known to all; the pain is frequently exquisite, and as the biliary fluid can find no exit, and its secretion still goes on, a reflux into the gall-bladder takes place, causing its distention and thus forming a tumor. In inflammation of the mucus lining of this duct, the cystic, and the gall-bladder itself, we also are enabled to discover a tumor by the touch, which will afford indications of increasing pain or pressure; while in the case of a mere mechanical obstruction, this will not be observed at least in so great a degree. The reason here is obvious; in their diseased conditions the vital actions are exalted, sensibility is increased, and pain is the consequence.

The situation of the tumor formed by the distended gall-bladder, will generally be found immediately under the cartilaginous border of the ribs, in the position which it naturally occupies; but when the augmentation is great, it may be felt lower down in the right hypochondrium; and some cases are on record, where it has occupied a portion of the epigastric region, or reached to within a short distance of the iliac crest. Where the swelling is very perceptible, independently of the aid to be derived from the occurrence of jaundice and whitish stools, we may be much assisted in the diagnosis, by ascertaining that the liver itself does not pass beyond its proper limits; and percussion too, yielding a sound not so flat as it would do, were this organ the point upon which it was made, offers an additional ground upon which to form an opinion. A circumstance peculiarly characteristic, of an enlargement of the gall-bladder, ought not here to be overlooked, and it is in respect to its *form;* the tumor in the two cases which we have ourselves observed, and in several which we have seen reported, is always *performed,* whatever may have been its state of distention. In one case where the gall-bladder was itself the point of lesion, it was exceedingly sensible to pressure; while in the other, depending upon a temporary obliteration or obstruction of the common, and possibly of the cystic duct, it was movable and indolent.

We shall not in this place attempt to point out all the alterations of texture which the various ducts, as well as the receptacle of the bile itself may undergo; as we have said above, some are not clearly discernible until after death; and it is only by inference that we can form an opinion during life. It may be remarked, however, that the inflammation of the mucus tunic of the ducts and gall-bladder, may terminate in ramollissement, ulceration, and finally perforation. This last complication, should an adhesion not have been formed with some portion of the intestine, is probably always productive of a fatal peritonitis.

This part of our subject we cannot dismiss without saying a word or two relative to the treatment in hepatic affections; and what is it? Why, in this country and in England, the moment a yellow sclerotic and whitish evacuations are observed, all the changes are rung upon the various combinations of *mercury* and its different modes of administration. The bowels must be purged, for they are slow to act, and they require the stimulus of the bile which nature has provided for them: and as a substitute we throw in twenty grain doses of calomel, and proportionate quantities of jalap, and rheubarb, and colocynth; with these remedies we fail, and we resort to muriatic acid internally, to nitro-muriatic baths, and to dandelion, and still we fail; and finally we arrive at the conclusion, that to the incurability of the disease must we attribute our ill success. True, the disease is incurable; but have not these very remedies rendered it so? They have in our opinion contributed to excite still further the already actively inflamed mucus membrane of the ducts, the gall-bladder, or the parenchyma of the liver itself; and thus they tend to increase and prolong the affection they were expected to alleviate. But let us act rationally upon this subject, the true principle of which is to remove by agents purely anti-phlogistic, and not attempt it by exciting cathartics, the stage of active inflammation. By these means we shall proceed with greater security, and when once the inflammation is subdued, the bowels return to their proper condition, and the sclerotic to its natural whiteness. The first case of inflamed gall-bladder which we witnessed, was characterized by both functional and local symptoms, and how speedily did it yield to moderate venesection, to leeches to the anus, and to cataplasms! We do not mean by what we have said, entirely to proscribe the use of mercury; but the state of acute phlogosis to which we have alluded, is not the one wherein it can be beneficial."

I have said that the writer seemed to place too much confidence in percussion when speaking of hepatic lesions. The same observation applies to his inquiry into the pathology of other organs. It would require an experienced ear to ascertain whether tumefaction of the epigastrium proceeded from schirrous of the stomach, tumors attached to its positerior or anterior surface, or even extensive hypertrophy of its walls. I am aware that fine distinctions between the resonance afforded on percussion, in the cases enumerated, may readily be drawn on paper, but when the physician is placed at the bedside of his patient the case is widely different. The same observations will apply to his remarks on uterine, ovarian, or mesenteric enlargements. He asserts that schirrous of the pylorus, can only be distinguished, from chronic gastritis, by the existence of tumor which is always present in the former affection. This may be true after the enlargement has proceeded to a certain extent, but in the early stages of the disease, when, only, there is a possibity of cure, no such tumor could be found.

The author seems to agree with Louis that typhus fever is invariably the result of an organic alteration in the glands of Peyer. This position may be true, but its truth is by no means confirmed. It requires a longer period as well as a greater diversity of observation to settle a question of so much importance. These glands, for instance, may be altered in their structure by the *cause* of the fever, or they may not have undergone any change in different climates from the continent of Europe. In order to prove that such lesion is the cause of typhus it must not only *always* be found in a single hospital or city, but it must also be detected in every country where the disease exists. Nay, even all this will not settle the question, for these bodies must be the only parts of the system where a lesion in *all cases of typhus* is found. Future discovery may reveal to us tissues which are affected equally as often as the Pyerian glands, and then the question will remain undecided which is the cause of the fever. I cannot, therefore, agree with the writer of the essay, when he asserts that "the unrivalled work of M. Louis, has incontestibly proved the true lesion of typhus to reside in an organic alteration of the glands of Peyer." The diarrhœa, which is said to be the result of ulceration of the glands so often named, is not always present in typhus fever. Cases of this disease terminate fatally without the presence of diarrhœa especially in the early or middle stages.

The greater part of Dr. Haxall's dissertation is taken up with an investigation of diseases of the thorax. I am much better pleased with this part of his paper than with that already noticed. It is true he has added but little to the discoveries of Bayle, Lænnec, Andral, Louis, etc., but he has collected and condensed much that was scattered through the works of various authors, and rendered the most important parts, accessible to the reader, without undergoing the labor of a lengthy investigation. His different kinds of respiration have already been enumerated by Lænnec, in his excellent work on the chest. Indeed it is questionable whether any writer will be able at a very early period, to contribute much to the physical signs of diseases of the thorax found in the works of Bayle and Lænnec. They may almost be said not only to have laid the foundation but also to have raised the superstructure and rendered it complete in all its parts. I cannot believe, however, that even in lesions of the chest auscultation and percussion will ever enable the great mass of the profesonis to determine the precise disease under which their patients are laboring. It requires much practice, as well as a discriminating ear, to render the stethoscope useful. Indeed, I have occasionally known those who professed to determine most by it, fail in their diagnosis, as examinations of the thoracic organs afterwards proved.

As I have presented my readers with the remarks of the writer on the liver, I will now lay before them what he has said in part on diseases of the lungs—the most interesting parts of the dissertation.

"Although the general and rational signs of pleurisy may lead us in a great many instances to recognize the true character of the lesion, yet are there cases of pneumonic inflammation, particularly in their inception, in which without the aid of physical signs, we may mistake the one for the other. We have encountered cases of pneumonia, where for the first few hours there was little or no expectoration; or if any, not of that peculiar kind which belongs to this disease, and in which the pain also, was as sharp and as acute as that of pleurisy. The face, too, has been seen to be without that flush of vivid redness, which has been given as one of the characteristics of pulmonary inflammation; and it must be admitted by all who have had much experience in the two diseases, that it would be impossible to look for a pathognomonic sign in the condition of the pulse. Varying as this must do by so many circumstances connected with the individual, founded upon idiosyncracy and the different susceptibilities in the chain of sympathies, it would be vain to draw a comparison so well founded as to serve as a sure and invariable guide. Nor is this all; pleurisy occurs as a secondary lesion in some diseases, and may really become the efficient cause of death; and that too, under circumstances where its rational symptoms would rarely, if ever, point out its existence; besides, it is only by the physical signs that we can distinctly trace the various changes which supervene during its progress, and mark unerringly the amount of effusion and its gradual diminution. And it is within the experience of those who have had the amplest opportunities for observation, that all the local symptoms, as pain, dyspnœa, etc., may fail, while yet the physical signs may exist in their fullest extent. Latent pneumonia, too, giving rise as it does to but feeble functional indications, yet sufficiently so to authorize a belief in *some* thoracic lesion, will almost always leave the uninitiated in physical signs in doubt and uncertainty.

As we have done in relation to all the diseases hitherto investigated, so shall we still refrain from entering upon a full history of the general and functional symptoms of those yet to be discussed. Indeed, they will only be mentioned when it becomes necessary to state their insufficiency towards the formation of a clear diagnosis, comparatively with the physical signs; and as the remarks which have just been made relative to inflammation of the pleura and the pulmonary parenchyma, may induce at least some little doubt as to the value of the functional signs in *all* cases, we will proceed to enumerate those, which from their nature cannot leave us in error.

Where the physician is called to a case of pleurisy during the first moments of its invasion, he might probably be at a loss in his diagnosis, except he were to reason upon the principle of exclusion; the signs of all other affections of the lungs or pleura being absent, he might reasonably infer the existence of this. But he would not long remain in doubt; for it is often surprising with what rapidity effusion takes place into the cavity of the pleura, while the inflammatory orgasm in its highest degree may yet remain unsubdued. Lænnec tells us that he has occasionally discovered effusion within one hour after the commencement of the disease, oftentimes within the space of three or four hours, and that it is never doubtful after the second day.

As soon as the effusion does take place, there can be no further room for doubt. The lung crowded towards the spinal column by the pressure of the fluid, permits the respiration to be heard only along a portion of its course, and that to the extent of some two or three fingers' breadth; above the spine of the scapula, unless the effusion be very great, and beneath the clavicle, it may also be discovered. This absence of respiration is owing of course to the temporary obliteration of the air-cells and minute bronchial ramifications, in consequence of the pressure exerted upon them; and it is only because these last are sufficiently firm and large at their root or origin to offer the necessary resistance, that the respiration there remains audible. The lung of the diseased side being thus deprived of its power of receiving air, the opposite lung, in order to make amends as it were for the deficiency of its fellow, appears to undergo an increase of action, and the respiration assumes the character denominated *puerile*. This when very loud and well marked, as it sometimes is, may lead the inexperienced into error; for it may be heard when the instrument or ear is applied to the diseased side, the effusion not being excessive, and thus impose the belief that the lung is here still permeable to the air. The diagnosis might then be called in question; and unless the difference between puerile and bronchial respiration was very clearly understood and appreciated, it might even be imagined that inflammation of the pulmonary parenchyma existed; the dullness of sound upon percussion would also aid such a supposition, and if ægophony be at the same time mistaken for bronchophony, as is not unfrequently the case, persistence in the error would be still more certain. But he who has once heard the full, hard, and noisy sound of bronchial respiration in pneumonia, and that too passing immediately *beneath* his ear, can hardly mistake it for the loud though soft vesicular murmur of infancy; an attentive examination in the case before us, will give the sensation too of the sound coming from a distance, which as has just been intimated, is not the case when the pulmonary tissue is inflamed. It is not pretended that this state of things is of frequent occurrence; on the contrary it is very rare; the respiration being in a large majority of instances, nearly if not quite inaudible on the affected side, except at the root of the lung.

When the condition of the thoracic cavity is unaffected by disease, the application of the hand upon its sides, during the process of respiration, and more particularly the act of speaking, imparts to it a peculiar trembling sensation; and if we use percussion over any of its regions, a well marked resonance. greater in some situations than in others, for obvious reasons, is uniformly elicited. The mechanism of both phenomena is easy of explanation ; the permeable parenchyma, dilating and distending itself under the influence of the introduction of the air, comes closely into contact with the sides of the chest, and the voice reverberating throughout the narrowest recesses of the pulmonary apparatus, thus causes the motatory and trembling feeling which is appreciated by the touch ; and the same anatomical character of the lungs to which allusion has just been made, viz. their permeability, although not rendering them hollow organs in the strict acceptation of the term, even when conjoined with the fact that numerous canals pass in every direction, yet makes them enough so to produce a clear resonance on percussion. When effusion has resulted, a denser medium being thus substituted for the air, which no longer finds admission into its appropriate cells in consequence of compression of the lung, the thorax loses the resonance which belongs to it in its natural state, and becomes dull when percussed.

Another physical sign, which is only observed while effusion is present, is ægophony: nor is it then heard, unless the effused fluid be moderate in quantity. From this fact we are therefore always enabled to tell, if no other symptom existed, whether the effusion be excessive or not ; if it be so, this sign, as we have remarked, will not be heard, and should it afterwards be recognized, an affirmative and positive indication of its diminution is established. In order to appreciate the reasons upon which these remarks are founded, it will be necessary to enter into an explanation of the production of ægophony. This sign bears some resemblance to bronchophony, and it does so, because a similar condition of the lung in part, is required to produce both. This condition is its greater density. When it has become more dense than natural, its pathological condition it is true is not the same, although its effect upon the development of the two signs alluded to, may be alike. In the one case, (that of bronchophony) the parenchyma is engorged, overloaded and impacted with blood, and the molecular or ultimate order of the tissue; is obviously deranged. In the other, the density is merely the result of compression, and no other change takes place in the organic structure, than a closer approximation of its parts. This dense or compact state of the lung being established, and the result of either the one or the other cause, prevents that diffused resonance of the voice which is noted in its healthy condition, as to the part affected we mean; and as all more solid bodies are better conductors of sound than those less so, the voice is heard within the chest with a distinctness proportioned to the solidity. Were there no effusion, the increased density of the lung, or a portion of it, as the case might be, from whatever cause it might arise, would produce pure bronchophony ; but while it exists, if moderate, the resonance of the voice is so modified by the medium through which it is heard, (the effused fluid,) that a certain trembling sensation, a sort of saccade, is distinguished, which imparts to the sign of ægophony its characteristic peculiarity. And from this explanation we at once see why it requires but a moderate quantity of fluid to allow of its detection; if it be excessive, the medium through which the resonance would have to pass, opposes an insurmountable barrier to its appreciation.

Ægophony is one of the physical signs to which we may look with very great certainty in almost all cases of pleurisy, and from what has been said it may be inferred that the stage of the disease in which it is most generally heard, is that wherein absorption of the fluid has taken place to a considerable extent. But it occasionally happens, though rarely, that it is heard throughout its whole continuance; and this is owing, as dissection has proved, to the lung being held near the sides of the chest, by adhesions which had formed in consequence of some former attack. During the early part of the disease, ægophony is sometimes met with, before the effusion has made sufficient progress to impede its occurrence.

Another sign, the consequence of effusion, is dilatation of the affected side, and this is frequently observable a few hours after the fluid is secreted; because there are cases in which it at once becomes very considerable. The ribs appear to be elevated, the intercostal spaces are augmented in breadth, and in proportion to the excess of the fluid after it has reached a certain point, is the diaphragm depressed: and hence it is that very often a deceptive appearance of enlargement of either the spleen or liver is observed, and more particularly the latter, on account of its superior volume. It is undoubtedly the chronic form of the disease, in which dilatation is more uniformly perceptible, and the phenomena to which we have alluded are more distinctly marked. Although always commencing early, it may not be clearly distinguishable for some little time, yet it ultimately increases to such a degree as no longer to be mistaken. All the physical signs hitherto mentioned, except ægophony, are eminently developed, and this for the reason already given. The lung, in consequence of protracted compression, appears to be nearly destroyed; its tissue is pale and exsanguineous, and its vessels and bronchial tubes are flattened. The character of the effusion is also found to vary; it is often purulent and is of a yellowish color. In this state the disease is denominated empyema.

Should life continue sufficiently long, and the absorption of the fluid take place, the affected side becomes contracted in its dimensions. This is the result of the slow and gradual manner in which adhesions are developed; and did it belong to us in this place to enter minutely into the mode of their formation, we might offer an interesting history of the process. It is sufficient for us to say here, that they ultimately assume a fibro-cartilaginous structure, and thus tend to bind down and constrict the sides of the chest. In the meantime, the lung itself remains compressed and flaccid. The signs of this complication are either a total absence of respiration, or it is but slightly heard and that near the root of the lung or in its superior portion. The thickness of the accidental developments does not cause this indistinctness or absence of the respiratory murmur, as the case may be, and which might readily be inferred from a hasty examination. Dissection reveals the true cause, by exhibiting the lung in a compressed condition, and thus inhibiting the ingress of the air into its cells; and the same pathological feature will abundantly account for the dullness of sound on percussion, which presents itself as another sign.

Before effusion takes place, or even while it is yet progressing, and we refer now to the more acute form of pleurisy, a thick, tenacious and plastic exudation lines that portion of the pleura which is in a state of inflammation. This is the first form of false membrane, or accidental tissue as it is called, and it is a curious circumstance in the history of this affection, that the hitherto unaffected portion of the pleura opposite to the diseased part speedily itself becomes inflamed; false membrane is here also produced, and we refer to this condition more particularly for the purpose of explaining another physical sign which is not very uncommon. In a state of health, the serous surfaces of the pleura, bedewed with a thin and slightly viscous fluid, glide easily and imperceptibly over each other. But so soon as this false membrane is exuded, the situation of things becomes altered, and in proportion to the degree of consistency which it assumes, by so much is the free and easy movement of the opposite surfaces, the one upon the other, impeded. Rough surfaces are opposed where before they were perfectly smooth, and if the ear or the instrument be applied, the sound elicited by their friction is easily distinguished. This has been termed the ascending and descending friction sound, the former being produced during inspiration, and the latter during expiration. We need scarcely remark that this sign is only appreciable either before the existence of the secreted serosity, or after it has been absorbed.

There is such a disease as partial or circumscribed pleurisy, and its modus formationis will very succintly be detailed. It is not of very frequent occurrence, because experience has proved the fact that in a pleura already adherent, inflammation supervenes more rarely than where this is not the case. Partial pleurisies are those circumscribed by former adhesions, and rarely if ever exist under other circumstances, except in two cases which will be mentioned under the heads of pleuro-pneumonia and phthisis. They are generally found in the fissures located between the lobes; between the base of the lung and diaphragm,—upon that part of the pleura covering the posterior and inferior portions of the lung, and between it and the mediastinum. The physical signs are absence of respiration in the part affected, occasionally ægophony is manifested, and where the situation of the diseased part will admit of percussion, a flat or dull sound results."

With the exception of a few errors, of either composition or typography, the work will bear comparison, in a literary point of view, with similar productions in any country.

